# Estimation of efficacy early selective LPS sorption in patients with septic shock

**DOI:** 10.1186/cc11762

**Published:** 2012-11-14

**Authors:** VV Kulabukhov, AN Kudryavtsev, AG Chizhov

**Affiliations:** 1 Vishnevsky Institute Of Surgery, Burn Center, Moscow, Russia

## Background

Recent improvements in the treatment of severe Gram-negative sepsis have not resulted in a substantial decrease in mortality. LPS, originating from Gram-negative bacteria, is a major mediator in the development of septic shock [1]. Decreasing the load of LPS in these patients may be beneficial for these patients. The Alteco LPS Adsorber^® ^selectively binds the lipid A moiety of LPS. Positive effects of the adsorber have been reported [2-4].

## Methods

Nineteen patients with Gram-negative sepsis were treated with the Alteco LPS Adsorber^®^, in addition to standard therapy according to SSC Guidelines 2008. All patients needed inotropic support and mechanical lung ventilation. The mean APACHE II score at the start of treatment was 25.9 ± 1.8. The time to initiating the perfusion varied between 1.7 and 8.6 hours after the diagnosis of septic shock. The treatment lasted 120 minutes and was repeated after 24 hours. The SOFA score, oxygenation index (PaO_2_/FiO_2_) and the dose of dopamine was noted before and 48 hours after the treatment.

## Results

**Figure 1:**
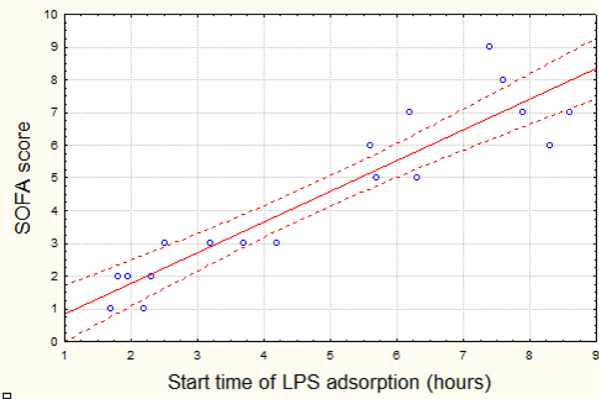
**Correlation between the SOFA score after 48 hours observation and the time of LPS adsorption start**.

**Figure 2:**
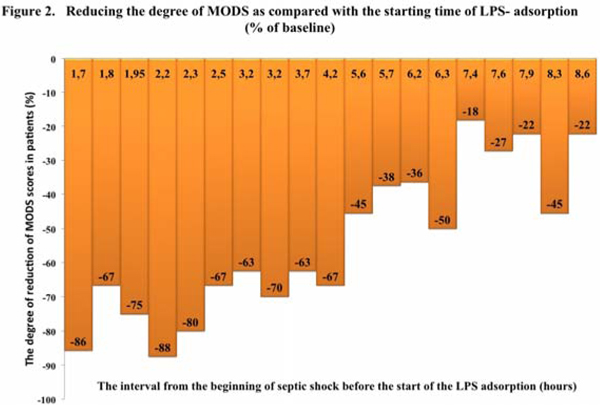
**Reducing the degree of MODS as compared with the starting time of LPS adsorption (% of baseline)**.

At baseline, the severity of MODS was SOFA score 9.2 ± 2.8. At 48 hours the mean score SOFA was 4.3 ± 2.7, at the expense of an increase in the index of oxygenation from 128.6 ± 36.2 to 253.5 ± 44.8; and a decrease in doses of inotropic support (dopamine) from 17.1 ± 1.8 mkg/kg/minute to 4.2 ± 1.8 mkg/kg/minute. We found an inverse correlation between the time to initiate treatment with the adsorbtion and the decrease in SOFA score (*R *= -0.69; *P *< 0.1) (Figure 1). The degree of reduction of MODS depending on time of initiation of therapy is shown in Figure 2. The 28-day mortality was 26.3% in this patient group. There were no adverse events related to use of the adsorber.

## Conclusion

Since LPS is a major mediator in Gram-negative sepsis, there is a rationale for rapid removal of LPS from patients with sepsis. Early initiation of LPS adsorption is most effective for reducing the signs of MODS. Our experiences with the use of Alteco LPS Adsorber^® ^are promising as shown by our data above and are in support of previous findings [2-4]. The results are limited by the lack of controls; however, we believe that further randomized controlled clinical studies using this therapy are warranted, more so in view of the fact that no specific treatment against septic shock is available.
